# Advancing horizons in vegetable cultivation: a journey from ageold practices to high-tech greenhouse cultivation—a review

**DOI:** 10.3389/fpls.2024.1357153

**Published:** 2024-04-15

**Authors:** Nazir Ahmed, Baige Zhang, Lansheng Deng, Bilquees Bozdar, Juan Li, Sadaruddin Chachar, Zaid Chachar, Itrat Jahan, Afifa Talpur, Muhammad Saleem Gishkori, Faisal Hayat, Panfeng Tu

**Affiliations:** ^1^ College of Horticulture and Landscape Architecture, Zhongkai University of Agriculture and Engineering, Guangzhou, Guangdong, China; ^2^ Key Laboratory for New Technology Research of Vegetables, Vegetable Research Institute, Guangdong Academy of Agricultural Science, Guangzhou, China; ^3^ College of Natural Resources and Environment, South China Agricultural University, Guangzhou, China; ^4^ Faculty of Crop Production, Sindh Agriculture University, Tandojam, Pakistan; ^5^ College of Agriculture and Biology, Zhongkai University of Agriculture and Engineering, Guangzhou, Guangdong, China

**Keywords:** seed treatments, soilless cultures, greenhouse technologies, precision agriculture, integrated pest management, digital monitoring

## Abstract

Vegetable cultivation stands as a pivotal element in the agricultural transformation illustrating a complex interplay between technological advancements, evolving environmental perspectives, and the growing global demand for food. This comprehensive review delves into the broad spectrum of developments in modern vegetable cultivation practices. Rooted in historical traditions, our exploration commences with conventional cultivation methods and traces the progression toward contemporary practices emphasizing the critical shifts that have refined techniques and outcomes. A significant focus is placed on the evolution of seed selection and quality assessment methods underlining the growing importance of seed treatments in enhancing both germination and plant growth. Transitioning from seeds to the soil, we investigate the transformative journey from traditional soil-based cultivation to the adoption of soilless cultures and the utilization of sustainable substrates like biochar and coir. The review also examines modern environmental controls highlighting the use of advanced greenhouse technologies and artificial intelligence in optimizing plant growth conditions. We underscore the increasing sophistication in water management strategies from advanced irrigation systems to intelligent moisture sensing. Additionally, this paper discusses the intricate aspects of precision fertilization, integrated pest management, and the expanding influence of plant growth regulators in vegetable cultivation. A special segment is dedicated to technological innovations, such as the integration of drones, robots, and state-of-the-art digital monitoring systems, in the cultivation process. While acknowledging these advancements, the review also realistically addresses the challenges and economic considerations involved in adopting cutting-edge technologies. In summary, this review not only provides a comprehensive guide to the current state of vegetable cultivation but also serves as a forward-looking reference emphasizing the critical role of continuous research and the anticipation of future developments in this field.

## 1 Introduction

Advancing horizons in vegetable cultivation signifies a critical evolution from traditional, soil-based methods to innovative high-tech greenhouse cultivation. This shift, pivotal in agricultural methodologies, represents a fusion of sophisticated technologies, enhanced understanding of plant biology, and a commitment to environmental stewardship and sustainable practices. Central to ensuring global food security, this transformation in agriculture begins at the nursery, a crucial stage where the life cycle of young plants determines their future health, vigor, and yield (Mohanta, 2020). Historically rooted in empirical knowledge and tailored to local environmental conditions and cultural traditions, vegetable cultivation has now embraced a comprehensive approach involving meticulous seed selection, soil preparation, and strategic management of environmental factors and pests (Thomas et al., 2019). Facing increasing food demands from a growing global population and variable climatic patterns, the need to refine vegetable cultivation practices has become more urgent than ever. This journey, extending from seedling to harvest, transcends mere growth optimization; it is about developing resilient, sustainable, and efficient agricultural systems that can adapt to contemporary challenges (Yadav et al., 2018; Zarbà et al., 2019). Propelled by technological advancements, groundbreaking plant science research, and interdisciplinary insights, this evolution is revolutionizing vegetable cultivation techniques ushering in an era marked by precision and sustainability in agricultural practices (Dhen et al., 2019; Waiba et al., 2020).

The transition from a seed to a seedling represents a vulnerable and critical phase in the life cycle of a plant. The quality of seedlings, delineated by traits, such as vigor, root development, physiological health, and resistance to diseases and pests, has a profound influence on the productivity and health of vegetable crops (Gupta and Kumar, 2020; Sarraf et al., 2020; Zulfiqar, 2021). Advanced techniques in vegetable cultivation cater to optimizing these quality metrics. For instance, precision in watering enabled by advanced irrigation systems ensures neither overwatering nor underwatering, both of which can jeopardize young plants (Warner et al., 2018). Enhanced growth media, whether organic or soilless, can fortify root development and structure, ensuring efficient water and nutrient absorption, thereby promoting seedling health and vigor (Warner et al., 2018; Mariotti et al., 2020). Furthermore, the introduction of modern methods for detecting and managing pests and diseases can substantially reduce seedling mortality. Early detection, facilitated by imaging and diagnostic tools alongside the utilization of integrated pest management (IPM) strategies, can shield seedlings from potentially debilitating infestations (Ofuya et al., 2023). Additionally, as we grapple with the multifaceted challenges posed by climate change, dwindling arable land, and heightened pest resistance, there is a compelling and urgent need to explore and adopt innovative vegetable farming techniques. By nurturing resilient crops and minimizing resource wastage, advanced farming techniques can lay the foundation for a successful, sustainable, and productive cultivation cycle (Waiba et al., 2020; Ranganath et al., 2023).

This review offers a detailed insight into contemporary advancements in vegetable cultivation practices. As the agricultural sector evolves, driven by technology and increasing food demand, understanding these shifts is vital. This study will benefit farmers, researchers, and practitioners, aiming to streamline their practices. The historical context unveils the evolution of vegetable farming from traditional to modern techniques. Emphasis on seed quality, selection, and treatment underscores their role in optimal germination. The examination of soil and growing media touches upon innovations, such as soilless cultures and coir. Advanced greenhouse technologies, artificial intelligence (AI), and automation have revolutionized environmental control, whereas water management now emphasizes modern irrigation and moisture-sensing techniques. Nutritional management focuses on precision fertilization and organic nutrients, and pest control highlights IPM strategies and biological controls. This review covers the significance of plant growth regulators and stresses in modern transplanting methods and seedling acclimatization. Technological advancements, such as drones, AI, and imaging techniques, have been highlighted, along with the importance of sustainable vegetable farming practices. Finally, the review concludes by outlining future prospects and emphasizing the central role of research in steering the industry’s trajectory. This review serves as a concise guide capturing the essence of modern vegetable farming and its future direction.

## 2 Historical overview

### 2.1 Traditional methods of vegetable cultivation practices

**Figure 1:**
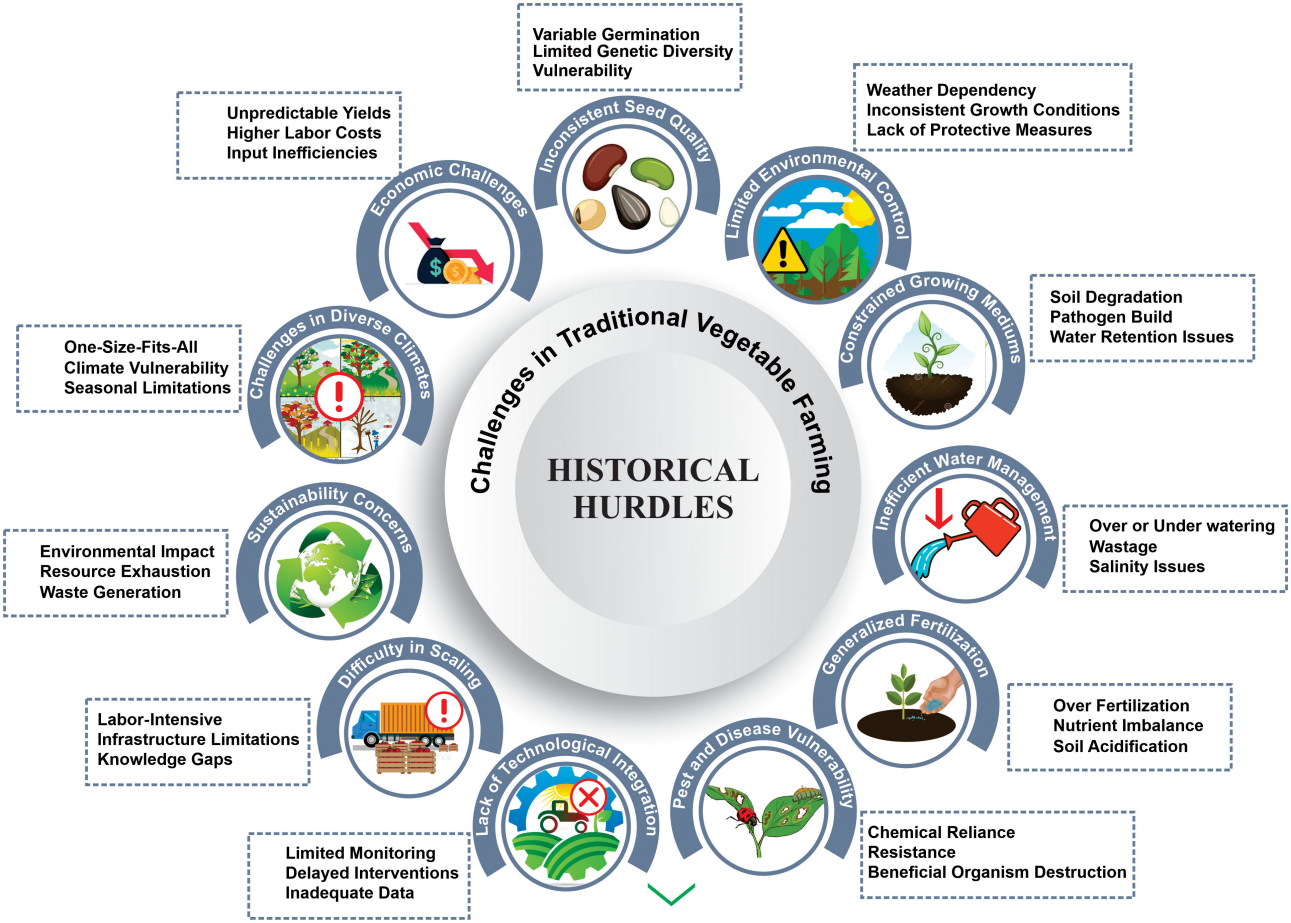
Multifaceted challenges in traditional vegetable farming: from seed quality to sustainability concerns.

Traditional methods in vegetable cultivation practices are deeply rooted in time-tested agricultural practices and sculpted meticulously by local environmental nuances and age-old cultural traditions. The emphasis on selecting seeds from the most robust plants underscores the primitive understanding of genetics (**Figure 1**). These plants, which are resilient to diseases and pests, are known to consistently yield superior produce (Reed et al., 2022). Seed treatments in this era were straightforward, yet effective. Common practices, such as soaking seeds in water or sun drying, aimed to enhance germination rates, a phenomenon detailed in the literature, such as that by Corbineau et al. (2023). In terms of soil preparation, this was an intensive hands-on affair. The ground was laboriously turned, weeds were removed, and any larger clumps were broken down to foster a nurturing environment for burgeoning seedlings. Organic matter, which predominantly decomposed farmyard manure, was meticulously mixed with the soil.

In regions susceptible to waterlogging, the landscape is often punctuated with raised beds, a strategy highlighted by Ayyam et al. (2019). Sowing is a meticulous process. Seeds were either broadcast by hand or sown in meticulously arranged lines, with each vegetable species dictating spacing to mitigate overcrowding and ensure robust growth. Traditional irrigation is, in essence, an exercise of simplicity and observation. The trade tools were rudimentary watering cans or basic hosepipes, and the cadence of watering was gauged by the keen eyes of farmers who watched for cues in soil moisture and crop vitality. Regions facing the challenge of intense sunlight or blustery winds witnessed innovation in the guise of temporary protective structures. Natural materials, such as straw or palm fronds, as referenced by Narvaez (2020), were repurposed to offer shade or counteract wind safeguarding vulnerable plants.

The solutions were predominantly organic when combatting pests and disease. Botanical remedies, such as revered neem oil or fiery garlic, chili, onion, and various other bio-pesticidal plant extract concoctions, are preferred (Baidoo and Mochiah, 2016; Benelli et al., 2017). Strategic agricultural choices, such as cultural methods, physical and mechanical barriers, sex pheromones, bio-pesticides and bio-control agents, and chemical and botanical means, have been employed as preemptive strikes against pest invasions, a sustainable approach explored in depth by Abhishek and Dwivedi (2021). The final rite of passage for seedlings, transplantation, was a meticulous endeavor. Upon reaching maturity, seedlings were gently uprooted and rehomed to the main fields, a process that demands finesse to ensure root and plant integrity. While these labor-intensive, indigenous knowledge-driven methodologies stood the test of time in their era, a relentless march of progress accompanied an era of modern agricultural innovations. This transition saw many traditional practices enhanced or replaced by mechanized and scientifically backed methods (Zhao et al., 2020). The ultimate goal of achieving unparalleled efficiency, consistency, and yields is to set the stage for the contemporary agricultural marvels we hold.

### 2.2 Evolution of techniques in vegetable cultivation practices over the decades

**Figure 2:**
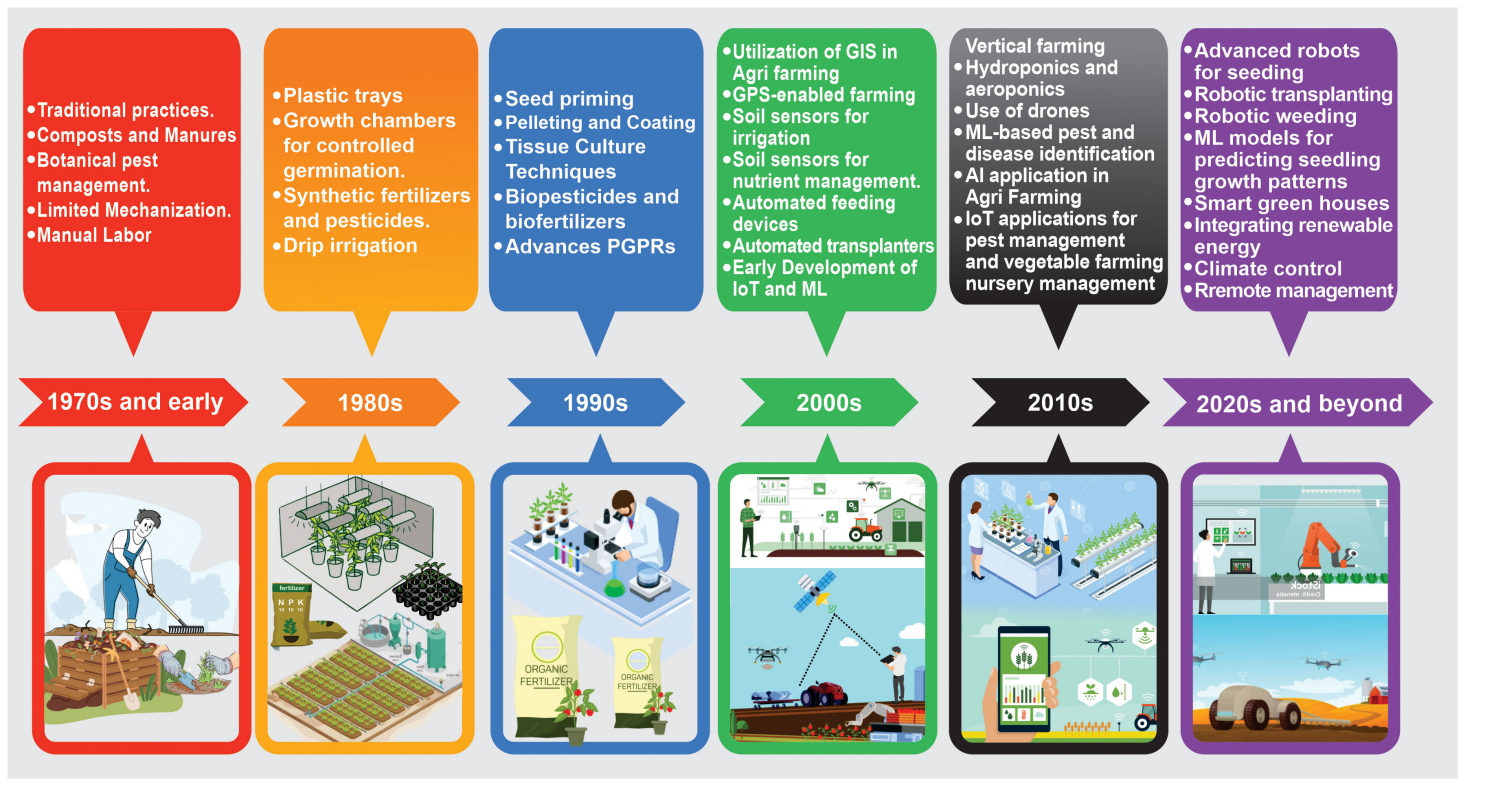
Chronological map of transformative techniques and technologies in vegetable cultivation practices from the 1970s through the 2020s. Spanning traditional practices to cutting-edge innovations underscores the rapid evolution of vegetable farming in response to changing agricultural needs and technological advancements.

Over the decades, the evolution of vegetable cultivation techniques has offered a fascinating glimpse into the broader arc of agricultural progression, characterized by technological innovations, deepening scientific insights, and a nuanced understanding of plant biology (**Figure 2**). Since the 1970s, vegetable cultivation has relied heavily on empirical knowledge passed down through generations. During this period, soil nutrition predominantly leaned from the organic compost and manure. Simultaneously, the pest management landscape is largely characterized by botanical solutions, with limited mechanization resulting in a predominant reliance on manual labor (Senesi, 1989; Benelli et al., 2017; Ofuya et al., 2023). The 1980s signaled a notable shift toward modernization especially in developed nations. This era witnessed the growing popularity of plastic trays for seed sowing and the introduction of growth chambers for enhanced germination control. As the agricultural sector expanded, so did the use of synthetic fertilizers and pesticides. An important breakthrough in this decade was the advent of drip irrigation, which promised efficient water management and marked a move toward precision agriculture (Xi et al., 2022; Yang et al., 2023).

By the 1990s, biotechnological innovations had begun to reshape the vegetable cultivation landscape. Seed priming, pelleting, and coating have emerged as novel techniques for ensuring improved seed vigor and consistent germination. The development of tissue culture techniques has been pivotal in enabling the mass production of disease-free plants. Although the foundational work in tissue culture dates back to the mid-1960s, it was not until the 1990s that these techniques were significantly expanded and refined to facilitate large-scale applications (Thorpe, 2007). Concurrently, there has been heightened emphasis on sustainability, with biopesticides and biofertilizers gaining traction as eco-friendly alternatives. Additionally, the role of beneficial microbial interactions in promoting plant growth has been explored (Gulzar et al., 2020; Liu et al., 2021; Raimi et al., 2021).

The turn of the millennium in the 2000s brought about an array of technological marvels. The integration of geographic information systems (GISs) with GPS-enabled farming tools marks a significant transformation in 21st-century agriculture revolutionizing farm management and decision-making processes. This synergy enhances efficiency and sustainability by combining the locational accuracy of GPS with the analytical prowess of GIS. Such integration allows for precise seedling placement and optimized irrigation practices, as evidenced by the deployment of soil sensors and nutrient management strategies (Nyakuri et al., 2022). Moreover, the advent of automation in vegetable farming, highlighted by the introduction of advanced feeding devices and transplanters, aligns with this technological evolution, further contributing to increased efficiency (Dadios et al., 2022). Notably, precision agriculture technologies, like GPS and GIS, have transcended traditional productivity measures offering in-depth insights into soil quality, crop distribution, and environmental conditions. These advancements not only bolster productivity but also reinforce sustainable farming practices, as they enable tailored interventions that cater to the specific needs of different farm sections (Koch and Khosla, 2003; Toscano et al., 2019; Nie and Yang, 2021).

The 2010s strengthened the agricultural sector’s commitment to sustainability and innovative farming practices. Vertical farming emerged as a solution to space constraints in urban settings, while techniques, such as hydroponics and aeroponics, revolutionized soilless cultivation (Zhang et al., 2018; Mustapha et al., 2022; Paucek et al., 2023). These advancements have been pivotal in addressing sustainability objectives by investigating water quality and microbial life in hydroponic cultivation contexts (Paucek et al., 2023). Furthermore, the period saw the increasing application of machine learning (ML)-driven diagnostics as essential tools for preemptive pest and disease identification highlighting the role of data-driven learning in agriculture (Tzounis et al., 2017). The burgeoning world of the Internet of Things (IoT) also saw application in real-time farm monitoring and management integrating advanced technologies to enhance agricultural efficiency and productivity (Ahmed et al., 2018; Raj et al., 2021; Namana et al., 2022).

Finally, the 2020s witnessed the emergence of more sophisticated technologies. Robotic systems have begun to perform various farm tasks, including seeding, transplanting, and weeding. ML models provide insights into crop growth patterns and optimized cultivation strategies. Gene-editing techniques, especially clustered regularly interspaced short palindromic repeats (CRISPR), have opened new avenues for improving crop traits. Smart greenhouses, integrating renewable energy sources, advanced climate control systems, and remote management capabilities, have become hallmarks of modern vegetable farming (Deshmukh et al., 2020; Fizikova et al., 2021; Kumar and Prabhansu, 2022; Zhu and Shang, 2022; Furquim et al., 2023).

## 3 From seed to seedling

### 3.1 Seed quality, selection, and advanced assessment techniques

**Figure 3:**
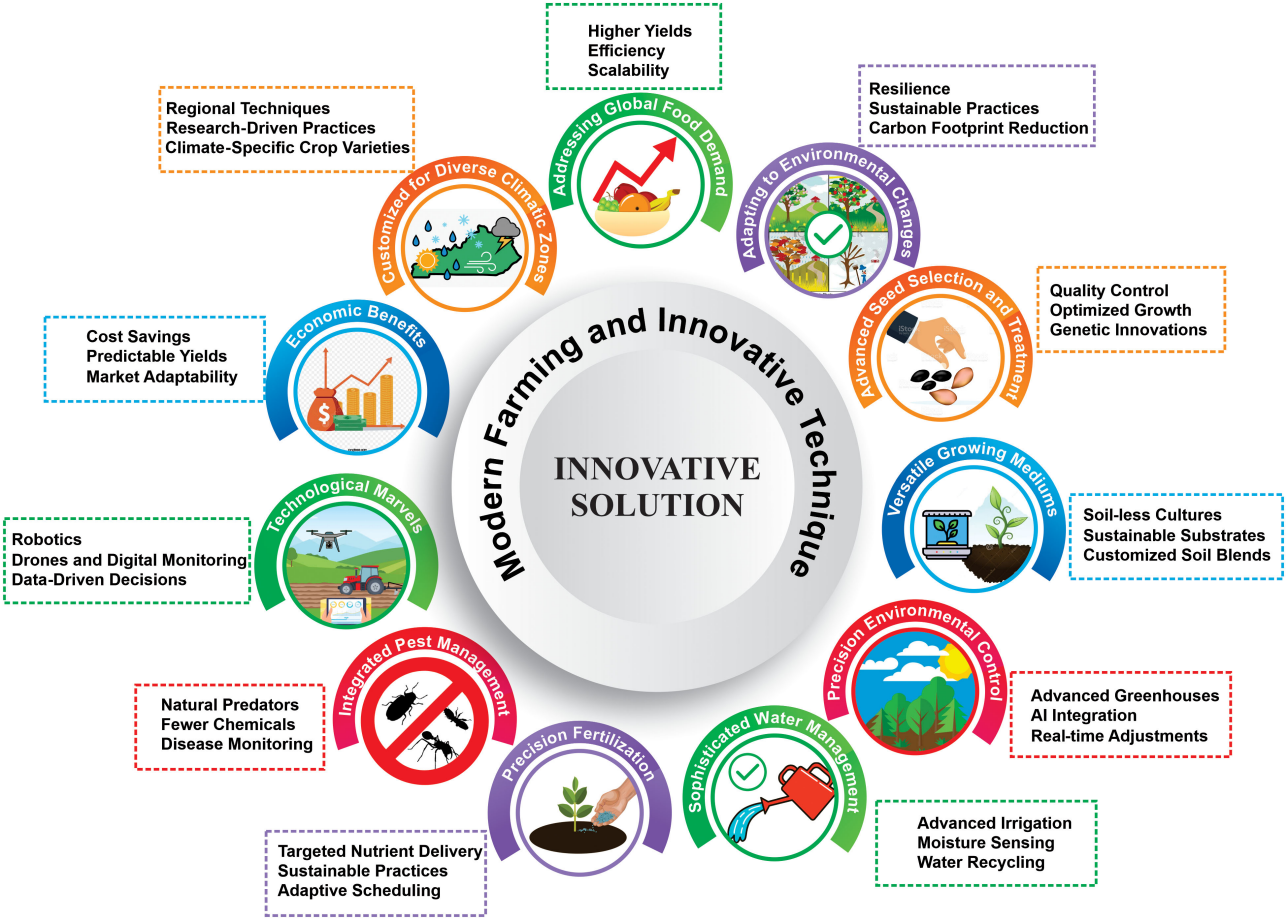
Revolutionizing agriculture: modern farming techniques and their multifaceted advancements for sustainable global food production.

The success of vegetable cultivation is fundamentally anchored in the quality and meticulous selection of seeds, a principle that has been an integral part of agricultural practices throughout history (**Figure 3**). The caliber of seeds sets the stage for the entire cultivation process dictating germination rates, uniformity in sprouting, and synchronized development across the crop. These attributes are critical for ensuring cohesive growth patterns and optimizing the agricultural yield (Pagano et al., 2023). Seed quality is a multifaceted concept that encompasses various attributes, including seedling vigor, which is a composite indicator of seed longevity, germination speed, and early stress tolerance. High-quality seeds are characterized by their ability to produce resilient seedlings capable of withstanding environmental adversities, such as fluctuating temperatures, drought conditions, and prevalent diseases, thereby contributing significantly to the overall robustness of the crop (Croft et al., 2018; Reed et al., 2022).

The genetic integrity of seeds is pivotal for ensuring the desired growth dynamics, resistance mechanisms, and yield potentials in crops. Understanding the metabolic pathways involved in stress responses, particularly the regulation of reactive oxygen species (ROS) homeostasis and DNA damage repair, is crucial for seeds to adapt to environmental challenges ( Schieber and Chandel, 2014; Waterworth et al., 2016). ROS serve as crucial signaling molecules in seeds influencing germination and stress adaptation by modulating metabolic and hormone pathways. The interplay between ROS and reactive nitrogen species (RNS) is essential for seed dormancy and germination particularly under stress conditions (Waterworth et al., 2016; Reed et al., 2022). ROS’s role extends to plant growth and development, where their balance is key; while they support acclimation to stress and dormancy breaking, excessive ROS levels can lead to cellular damage ( Schieber and Chandel, 2014; Waterworth et al., 2016). The implications of seed quality are also economically significant and influence resource allocation and overall productivity. Seeds of superior quality allow for the optimized utilization of water, growing medium, and labor, thereby minimizing waste and maximizing returns. Innovative management programs, such as the Integrated Knowledge and Products Strategy (IKPS), have demonstrated that the application of quality seeds, in conjunction with efficient farming practices, can lead to substantial reductions in resource use and environmental impacts while increasing yield (Wang et al., 2021). Investment in quality seeds provides a robust foundation for successful cultivation promising not only higher yields but also greater environmental and economic sustainability.

The assessment of seed quality has evolved significantly, transitioning from reliance on traditional, empirical methods to the adoption of advanced, scientific techniques. Historically, seed selection was predominantly based on visual inspection, focusing on attributes like size, color, and texture. For example, manual sieve analysis has been employed for grading seeds, such as chickpeas, based on their size (Khatri and Agrawal, 2021). In the modern era, technological advancements have revolutionized seed quality assessment, introducing non-destructive, efficient methods such as optical and multispectral imaging. These cutting-edge techniques allow for a rapid and comprehensive evaluation of seeds’ physiological potential and vitality enabling the detection of mechanical damages and other imperfections that are not visible to the naked eye (Salimi and Boelt, 2019; Galletti et al., 2020). Complementing these imaging techniques are biochemical assays, including tetrazolium and TTC tests, which provide insights into the metabolic activity and viability of seeds. These assays can be tailored to specific seed types by adjusting parameters, like exposure times and chemical concentrations, offering a versatile tool for seed quality assessment (Salazar Mercado et al., 2020).

Germination tests remain a cornerstone of seed quality assessment serving as a fundamental and reliable method to gauge seeds’ potential to develop into healthy plants. These tests often validate findings from more rapid assays ensuring a comprehensive evaluation of seed quality (Reed et al., 2022). Moreover, innovations, such as X-ray analysis, have opened new vistas in seed assessment allowing researchers and practitioners to peer into the internal structure of seeds. This technique is particularly valuable for identifying internal damages or defects that could impact seed viability and, subsequently, crop yield (MaChado et al., 2020). The genetic integrity of seeds is another critical aspect of seed quality with molecular markers and next-generation sequencing playing pivotal roles in ensuring genetic robustness. These techniques are instrumental in preserving genetic diversity and safeguarding against genetic drift or contamination, thereby maintaining the desired traits in cultivated crops (Salgotra and Chauhan, 2023).

Additional methodologies, like electrical conductivity tests, offer unique insights into seed vigor by quantifying electrolyte leakage providing a proxy for membrane integrity and overall seed health (Ortiz et al., 2018). Pathogen detection has also seen significant advancements with techniques, such as PCR coupled with nanomaterial-based sensors, enabling rapid, accurate identification of seed-borne pathogens ensuring the sowing of healthy, disease-free seeds (Kumar et al., 2021). Hence, the modern approach to seed quality assessment in vegetable cultivation is characterized by a harmonious blend of traditional wisdom and advanced scientific innovations. This comprehensive strategy not only facilitates the selection of superior-quality seeds but also ensures the resilience, health, and sustainability of future crops meeting the complex demands of contemporary agriculture and contributing to global food security.

### 3.2 Seed treatments and their impact on germination and seedling growth, vigor, and stress tolerance

Seed treatment plays a critical role in enhancing seed quality, optimizing germination rates, and fostering the health of emerging seedlings. These treatments, as illustrated by Ahmed et al. (2019) and Sharma et al. (2015), ensure faster and consistent germination while enhancing resilience against various biotic and abiotic challenges. One primary treatment, seed priming, involves controlled hydration of seeds. Pre-germinative metabolic activities are initiated by soaking seeds in either pure water or specific solutions. This process imbues seeds with vigor leading to resilient seedlings that can better navigate environmental challenges (Raj and Raj, 2019). In addition to priming, seed coating and pelleting have gained prominence. Seeds are enveloped by materials ranging from organic clays to modern synthetic polymers. Some treatments integrate advanced elements, such as nanoparticles, for additional protection. For example, zinc nanoparticles (Zn NPs) used in seed treatments are invaluable given their role in the synthesis of enzymes crucial for germination. Appropriate concentrations of ZnO NPs can enhance germination and vigor (Timilsina and Chen, 2021). As for stress tolerance, seed treatments have been especially vital in countering drought stress (Ahmed et al., 2019). Research on plants, such as *Vicia faba*, indicates that seed priming with specific extracts can trigger genetic adaptations to mitigate the adverse effects of drought (Kasim et al., 2019). Another promising strategy is the application of plant hormones. Hormones, such as gibberellins (GAs), cytokinin, and auxins, can expedite germination, deter pests, and mitigate oxidative stress, thereby leading to healthier plants (Ghafoor et al., 2020; Madany et al., 2020). GA_3_ priming enhanced germination and early growth of rapeseed under drought conditions simulated by PEG-6000. These data suggest that the decreased germination and growth in non-primed seeds during drought are due to increased ROS production. Seeds primed with GA_3_ showed increased activity of antioxidants, such as superoxide dismutase, peroxidase, catalase, ascorbic acid, and glutathione (Zhang et al., 2022). In certain crops, such as lettuce, seeds primed and then coated with clay-based materials can germinate efficiently even under elevated temperatures, a boon for hotter climates (Valdes and Bradford, 2022). Biopriming offers another innovation avenue. Treating seeds with beneficial microorganisms can enhance germination and overall plant growth. For instance, a blend of certain beneficial microbes proved to be more effective than traditional fungicides in French bean (Negi et al., 2021). Thermotherapy, which subjects the seeds to specific high temperatures, can neutralize seed-borne pathogens, while introducing beneficial microbes to seeds improves nutrient absorption and provides a natural defense against diseases (Ding et al., 2013; Davino et al., 2020). Furthermore, addressing threats, such as the tomato brown rugose fruit virus (*ToBRFV*), involves understanding its presence in seed parts and evaluating different disinfection methods. Chemical pesticides, while effective against pests, such as nematodes, require careful application to preserve seed integrity during germination (Patil et al., 2021). In essence, seed treatments equip seeds for their forthcoming journey, ensuring that they are not only ready but primed for excellence, laying the groundwork for prolific harvests.

## 4 Role of hardening, acclimatization, and transplantation

In vegetable cultivation, the convergence of traditional practices and advanced methodologies has been prominently demonstrated in the techniques of hardening, transplantation, and acclimatization (Melissas et al., 2022). Young seedlings, although brimming with potential, face inherent vulnerabilities in their formative stages (Bag et al., 2019). The hardening process, which entails gradual exposure of young plants to fluctuating environmental conditions, strengthens them against potential stresses (Bag et al., 2019). Transplantation, on the other hand, aids in their movement to more conducive habitats optimizing root development and plant establishment (Shao et al., 2021). Concurrently, acclimatization acts as a pivotal transition assisting young plants in adapting to sheltered environments, such as greenhouses, and to the vagaries of open terrains (Bag et al., 2019). Collectively, these methods exemplify the synergistic blend of age-old agricultural insights and contemporary vegetable farming practices emphasizing the importance of fostering crop resilience and vitality.

### 4.1 Seedling hardening and acclimatization

Seedling hardening, a revered tradition in agriculture, is now viewed through the lens of both conventional wisdom and state-of-the-art methods. This phase seeks to bolster seedlings getting them field ready by enhancing their resilience against potential environmental adversities. In the past, hardening was achieved via simple tactics such as modulating water intake and progressively introducing seedlings to sunlight. However, in the modern era, seedlings are immersed in nutrient-rich solutions that offer vital nutrients, such as nitrogen, phosphorus, and potassium, setting them on a path of vigorous growth and efficient energy transfer (Marschner, 2011). However, the growth story does not end with basic nutrients. Growth regulators, such as auxins and cytokinins, are fundamental in refining root development and cellular division. This adaptability enables crops, such as strawberries, to overcome hurdles, such as soil salinity, resulting in an improved photosynthetic rate and better cell structure (Zhang et al., 2021; Padilla et al., 2023). Adapting to the environment’s whims is critical. Techniques, such as cold hardening, saline conditioning, and controlled water stress, are pivotal. For instance, cold hardening introduces seedlings to reduce temperatures ensuring resilience against unexpected frost (Kolupaev et al., 2020). The mutualism between seedlings and beneficial microbes has also been highlighted. For instance, mycorrhizal fungi not only deter diseases but also boost nutrient absorption (Aini et al., 2019). Simultaneously, the integration of compost with such microbes has increased drought resistance in crops such as tomatoes (Tahiri et al., 2022). There is growing interest in anti-stress compounds. For example, osmoprotectants counteract the negative impacts of environmental stressors (Ahmed et al., 2021a). In onions, proline has been a game-changer for mitigating drought-induced stress (Semida et al., 2020).

The integration of controlled UV hardening and specific light spectra in seedling development has shown significant promise in enhancing plant growth and resilience. UV hardening, involving exposure to UV radiation doses, stimulates phenolic compound production, a critical factor in plant defense mechanisms (Strid et al., 1994; Teklemariam, 2002). Advancements in light-emitting diode (LED) technology have led to the development of phosphor-converted (PC) white LEDs, which utilize a phosphor layer over blue LED chips to produce white light (Zhao et al., 2019). This process creates a broad spectrum, predominantly in the green–yellow–red range, and is known for its high energy efficiency and cost effectiveness due to mass production (Meyer and Tappe, 2015). However, for precise spectral needs, especially in enhancing plant growth, systems with monochromatic LEDs, offering tailored red and blue light combinations, might be more beneficial (Kong et al., 2019). Monochromatic red and blue LED lights have been proven effective in improving dry shoot biomass, root architecture, and stem diameter of seedlings in both pre- and post-transplantation phases (Kong et al., 2019; Melissas et al., 2022; Zhao et al., 2023). Contrastingly, seedlings exposed to standard fluorescent lamps and isolated red or blue light have shown suboptimal growth especially under blue light (Zhao et al., 2023). The most beneficial light condition, particularly for high-quality grafted tomato seedlings adapting to transplant shock, involves a specific combination of red and blue light (Melissas et al., 2022). In certain artificial lighting configurations, light, primarily in the red wavelength range combined with a specific proportion of blue spectrum radiation, has been found to enhance stem diameter, root development, and the accumulation of phenolics and antioxidants. Furthermore, lettuce grown under alternating red/blue light demonstrated accelerated growth and higher levels of nutrients, like sugars, ascorbic acid, and anthocyanins, suggesting that this lighting regimen can enhance both growth and nutritional quality offering a dual benefit in plant factory settings (Ohtake et al., 2018). The transition to high-tech greenhouse cultivation has been greatly shaped by fluorescent lamps, whose wider spectrum emission offers more flexibility in light manipulation compared to traditional discharge lamps, marking a key advancement in controlled environment agriculture (Brown et al., 1995). Furthermore, the development and continued use of Growlux technology have further revolutionized *in vitro* plant cultivation exemplifying the technological progression toward optimizing light conditions for plant growth especially in contexts where natural light conditions are not sufficient or controllable (Morrow, 2008). The use of fluorescent lamps has been associated with increased MDA contents in shoots indicating their impact on plant growth and development (Astolfi et al., 2012). However, it is important to note that fluorescent lamps emit mercury, which can cause environmental pollution (Li et al., 2018). In contrast, LED lamps have been considered safer to operate and have lower energy consumption compared to fluorescent lamps making them an attractive option for controlled environment cultivation (De Carvalho et al., 2021). The effects of different light sources, including fluorescent lamps, LEDs, and high-pressure sodium (HPS) lamps, on plant growth and development have been extensively studied. For instance, the use of fluorescent lamps has been found to be effective for displaying flower colors and has been used as the standard condition in some studies (Yang et al., 2014). Additionally, the use of HPS lamps in greenhouse crops has traditionally been the main source of light in winter months, although LEDs are becoming more common in horticulture (Treder et al., 2021).

Additionally, the role of temperature fluctuations in preparing seedlings for varying outdoor conditions cannot be overstated. Such environmental control is crucial in the early stages of plant development for ensuring adaptability and resilience (Chalker-Scott and Scott, 2004; Indergard et al., 2022). Wind exposure also plays a major role in strengthening the seedlings (Gardiner et al., 2016). Biostimulants have earned their place in modern agricultural practice. For example, the application of *Ecklonia maxima* extract has shown dramatic improvements in plant growth, yield, and nutrient quality (Kocira et al., 2018; La Bella et al., 2021; Rakkammal et al., 2023). Physical manipulation, a seemingly simple approach, enhances stem strength proving that the most traditional methods sometimes retain their value (Bag et al., 2019). Blending traditional practices with contemporary techniques equips seedlings with strength and resilience paving the way for thriving agricultural landscapes.

### 4.2 Automated transplanting: modernizing vegetable farming practice

Transplanting stands as an age-old rite in agriculture, a practice that is steeped in tradition and skill. In its earliest form, transplanting was a manual, labor-intensive task that required an artisanal touch to mitigate root damage and prevent plant shock. With time and technological advancements, semi-automatic transplanters have emerged offering a perception of mechanization (Kacheyo et al., 2023). However, their efficiency often waned with operator fatigue leading to occasional inconsistencies in plant spacing and depth. This landscape has experienced a seismic shift with the dawn of automation in agriculture. Today’s automated transplanters, which merge cutting-edge technology with agrarian principles, promise both speed and precision (Khadatkar et al., 2018). These systems prioritize the delicate handling of plants, ensuring that root structures remain intact, and setting the stage for swift acclimatization in their new homes. By incorporating sensor technology, modern transplanters offer real-time feedback, which equips farmers with the ability to make instantaneous adjustments for optimal transplantation outcomes (Jin et al., 2019). Robotic systems with vision sensors or end effectors represent the pinnacle of this evolution. Individual seedlings were meticulously selected from trays to ensure precise placement at predetermined sites. Such systems typically combine an array of specialized components, such as vision systems, grippers, manipulators, and drum-type seedling removal devices, reflecting the harmony of precision and efficiency (Khadatkar et al., 2018). Research has underscored the multifaceted benefits of automated transplantation. Jin et al. (2019) emphasized the role of automation in minimizing transplant shock, which is a critical factor in determining seedling survival and subsequent growth. Furthermore, as Khadatkar et al. (2018) pointed out, automated systems have the potential to reduce operational costs in the long run while also consistently ensuring that seedlings are planted at optimal depths and spacings. Another compelling benefit, as highlighted by Christiaensen et al. (2021), is the potential reduction in labor shortages during peak planting seasons, which can profoundly impact crop yield and quality. The fusion of time-honored practices with technological innovations remains evident in this transformative journey from manual transplantation to advanced automation. Such advancements not only honor the essence of agriculture but also pave the way for a more efficient and sustainable future.

## 5 Soil and growing media innovations

### 5.1 Introduction to traditional soil and growing media

The success of vegetable cultivation is deeply anchored in the meticulous selection of the soil and growing media (**Figure 3**). Natural soil has historically been the cornerstone of vegetable cultivation sustaining agriculture for millennia by providing an organically rich habitat for plants. However, this reliance is not without challenges, including susceptibility to diseases, pest infestations, and potential nutrient deficiencies (Olle et al., 2012; Abd-Elgawad, 2019). Historical solutions to these challenges often involve the use of compost and other similar media. Compost, derived from decomposed organic matter, revitalizes soil by supplying a mix of nutrients and beneficial microorganisms while also enhancing soil structure (Zinati, 2005). As the 20th century dawned, the introduction of biocontrol agent-fortified composts amplified the disease-suppression capabilities inherent in traditional composts (Zinati, 2005). Peat moss, often paired with biochar, has become another popular growing medium lauded for its exceptional moisture retention and pH-balancing properties, which together create an optimum environment for root development (Bachmann et al., 2018; Vaughn et al., 2021). The course of growing media underwent a transformative shift with the advent of innovations, such as soilless cultivation methods, including hydroponics and aeroponics, which promised superior yields and expedited growth rates. Coir, a byproduct of coconut processing, has emerged as an eco-friendly alternative to peat moss (Mariotti et al., 2020). Concurrently, modern agriculture embraces bio-fertilizers, which introduce beneficial microorganisms to aid plants in nutrient uptake, thereby reducing their dependency on synthetic fertilizers (Fertahi et al., 2021; Raimi et al., 2021). Typical substrate components include minerals in natural or modified forms, such as sand, lava rock, expanded shale, clay, and slate; recycled waste materials, such as crushed bricks or tiles; crushed or aerated concrete and subsoil; and stabilized organic matter, such as composts, plastic materials, and slow-release fertilizers. Incorporating technology, sensors have begun to offer real-time data on the moisture, nutrients, and pH levels of the media, paving the way for precision agriculture. Leading-edge research has ventured into the potential of 3D printing and nanotechnology aiming to optimize and enhance the properties of growing media. Responding to the increasing demand for organic produce, organic-growing media devoid of synthetic additives made their mark. Furthermore, the emphasis leaned toward developing disease-suppressive media by integrating beneficial microbes consequently decreasing the dependence on chemical fungicides. Addressing specific plant requirements, the industry has witnessed a trend toward custom-designed growing media tailored exclusively for individual crops (Ampim et al., 2010; Przemieniecki et al., 2021; Antonious et al., 2023; De Marco et al., 2023). Drawing on the rich tapestry of time-tested approaches to soil and growing media, the current wave of breakthroughs and innovations signals a promising horizon. The continuous evolution of growing media, intertwining age-old wisdom with the forefront of scientific discovery, is poised to revolutionize agriculture addressing both the burgeoning needs of the global population and the pressing call for sustainable farming practices.

### 5.2 Advancements in soilless culture

Hydroponics, a novel technique for growing plants without soil, has significantly altered the landscape of controlled environment agriculture. Reports suggest that various aspects, such as dry matter, sugar, soluble solids, vitamins, and carotenoid content in crops, such as tomatoes, are superior when cultivated through soilless systems than in traditional soil (Olle et al., 2012). By replacing soil with nutrient-rich water, hydroponics offers numerous benefits, including increased nutrient absorption, faster growth rates, and elimination of many soil-borne diseases (Waiba et al., 2020). The journey began with basic hydroponics in which plants were grown in static nutrient-infused water (Maucieri et al., 2019). The convergence of hydroponics with recirculating aquaculture systems is a sustainable method for aquaponics. The choice of hydroponic technology in aquaponics depends on various factors, including environmental conditions, financial viability, crop type, and spatial availability (Maucieri et al., 2019). As this field progressed, innovative systems, such as the nutrient film technique, emerged, marked by a continuous flow of nutrient solution, making it especially useful for crops with shorter growth cycles, such as lettuce (Alipio et al., 2019). Another remarkable addition, deep-water culture, sees plants submerged in aerated nutrient solutions making it suitable for leafy greens (Nursyahid et al., 2021). Vegetable cultivation, from its primitive origins in foraging to the advanced vegetable science of today, has continually evolved. This discipline has not only developed high-yielding and nutritious vegetable hybrids adaptable to varying conditions but also devised techniques to counter climate adversities. These innovations extend to controlled environmental farming and strategies against climate change (Singh, 2023). Another advancement, aeroponics, maintains plant roots in the air intermittently misting them for nutrients. This approach maximizes access to oxygen, promotes growth, and reduces disease risk (Singh, 2023). Simultaneously, the drip system, utilizing intricate tubing and drip emitters, allows for the precise delivery of nutrients ideal for larger or specific plant species (Yang et al., 2023). Alternate growing media, such as coco coir and a blend of peat and perlite, although still considered hydroponic, have gained traction because of their impressive moisture retention and aeration capacities (Mariotti et al., 2020). Aquaponics, merging aquaculture, and hydroponics represent an epitome of sustainability, with fish waste providing nutrients for plants, which in turn purifies water for fish (Maucieri et al., 2019). Modern hydroponics is further augmented by technological advancements enabling automated monitoring and adjustment of parameters essential for plant growth, such as nutrient balance, pH, and temperature (Joshitha et al., 2021). Hydroponics, transitioning from its basic beginnings to a discipline enriched by technology, showcases the promise and potential of soilless cultivation in the future of global agriculture.

### 5.3 Importance of pH, electrical conductivity, and nutrient balance in growing media

The growth and prosperity of plants within a growing medium hinge on a myriad of interconnected conditions. The list of these essential factors is the pH level, electrical conductivity (EC), and overall nutrient balance present within the medium. Ensuring the optimization of these parameters is crucial for achieving peak plant health and yield. These findings have been consistently underscored across a range of studies affirming the foundational significance of these parameters in agriculture and horticultural farming systems (Marschner, 2011; Xiong et al., 2017; Fan et al., 2020). In greenhouse cultivation, maintaining the appropriate pH level of the substrate is crucial as it directly influences nutrient uptake by plants. A neutral pH level is marked at 7 on the scale with values below indicating acidity and above denoting alkalinity. Optimal nutrient absorption often requires precise pH adjustments to align with the specific needs of the plants being cultivated (Marschner, 2011; Xiong et al., 2017). Within intensive systems, such as plastic greenhouse vegetable production (PGVP), there is a risk of excess nutrients. Obsessive fertilization can trigger soil degradation compromising long-term usability. An illustrative study highlighted the nutrient surplus within PGVP, hinting at potential pitfalls, such as rapid soil nutrient build-up, acidification, and secondary salinization (Fan et al., 2020). EC is a pivotal benchmark for growing media. It represents the volume of dissolved salts, which is a proxy for nutrient concentration. EC media provide vital clues on whether plants are receiving optimal nourishment. Varying substrates, each with unique characteristics, can influence the EC, pH, and nutrient dynamics differently. Notably, as eco-consciousness rises, the quest for sustainable substrates has gained momentum. In this regard, alternatives, such as coconut coir, have shown promise rivaling traditional choices such as rockwool and peat vermiculite (Xiong et al., 2017). An ideal pH is slightly acidic for a diverse vegetable palette typically falling between 6.0 and 7.5. In vegetable-producing regions, critical water parameters, such as nitrate, phosphate, and total dissolved solid concentrations, require keen attention. The Best Management Practices strive to harmonize nutrient inputs and protect water sources while ensuring sustainable yields (Liu et al., 2022a). The pH spectrum intertwines with the soil microbial community impacting nutrient dynamics and bolstering plant health (Husson, 2013; Bárcenas-Moreno et al., 2022). The oxidation–reduction potential (Eh) is a dimension that is yet to be extensively studied. In addition to pH, Eh can reshape soil, plant, and microbial interactions. Groundbreaking research posits that an interplay between Eh, pH, and biological activity could revolutionize cropping strategies (Husson, 2013). Plant health is a nuanced ballet of multiple factors arranged within their growth medium with pH, EC, and nutrient balance as lead regulators. pH regulates nutrient gates, EC reflects nutrient content, and nutrient balance ensures collaborative functionality (Marschner, 2011; Husson, 2013). However, a misstep can disrupt homeostasis. As emphasized by researchers, these parameters are not mere numerical indicators, but play a crucial role in determining the overall performance and yield of crops (Bárcenas-Moreno et al., 2022; Liu et al., 2022b). Comprehending these elements gives one the ability to seamlessly orchestrate the entire growth process transitioning a plant from its nascent seedling stage to mature fruition. It is imperative to understand the nuances of pH, EC, and nutrient balance for those striving for maximum growth and yield. Based on this knowledge, growers can ensure optimal plant development and guide them to achieve their maximum potential.

## 6 Nutrition and water management in agriculture farming system: a vital shift toward sustainability

Thus, the importance of agriculture cannot be overstated. It not only feeds our burgeoning global population but also serves as a fulcrum for the ecological balance of our planet teeters. One of the linchpins of this balance is the nutrition management in agriculture (**Figure 3**). Traditionally, farmers have leaned heavily on broad-spectrum fertilizer applications. Although these methods deliver yields, they often result in significant over-fertilization, which, in turn, has a cascade of environmental repercussions. These include challenges, such as soil acidification, increased soil salinity, and the dire concern of heavy metal contamination, as detailed by Mikula et al. (2020). However, as pointed out by Tripathi et al. (2022), plants, much like humans, require balanced nutrition. This balance is a delicate dance of nutrients that, if skewed, can greatly affect plant health, growth, and resilience to diseases. Nutrients, such as nitrogen and potassium, have proven instrumental in bolstering plant defenses. Although nitrogen facilitates the amplification of defense-related enzyme levels, potassium plays a pivotal role in enhancing plant polyphenolic concentrations both of which are essential for defense mechanisms. Conversely, elevated phosphorus levels might increase the vulnerability of plants emphasizing the intricacy and delicacy of nutrient management.

Enter precision agriculture: a paradigm shift that could not have come at a more crucial time. Harnessing technological advancements, this approach, as elucidated by Bar-Yosef (2020), reported the precise provision of nutrients. This ensures that plants receive the exact quantities of nutrients they need. Such an approach not only optimizes yield but also dramatically reduces waste and mitigates the adverse environmental impacts associated with traditional farming methods. One of the most pressing areas of concern underlined by both Bar-Yosef (2020) and Mikula et al. (2020) is micronutrient management. Although indispensable, these nutrients can be toxic in excessive quantities. Metallic microelements, such as copper, iron, and zinc, if mismanaged, can concentrate in the root zone, which has detrimental effects on plants. Additionally, Tahat et al. (2020) elucidated the pivotal role of soil health assessment in nutrition management. A comprehensive understanding of soil quality, including the bioavailable forms of macro and micronutrients, paired with an assessment of weather conditions, can guide farmers in making precise decisions regarding fertilization (Rodríguez et al., 2022). This integrative approach ensures that the soil, the bedrock of agriculture, remains fertile and free of contamination. Facing the dual challenges of feeding a growing population and environmental conservation, advanced nutritional management in agriculture is crucial. Sustainable farming is now essential, and precision agriculture, which focuses on accurate nutrient allocation, offers a promising way forward showcasing the power of innovation for a sustainable future.

### 6.1 Organic nutrient usage in modern agriculture

In the contemporary age of agriculture, where sustainability is a clarion call, organic nutrients stand as a beacon for harmonizing farming practices with ecological necessities. Rooted in nature and eschewing the toxicity associated with many synthetic compounds, organic fertilizers embody the nexus between tradition and innovation. In natural cycles and ecological systems, organic farming hinges on the cyclical reintroduction of organic matter into the soil rejuvenating its fertility. This organic mantra transcends mere philosophical postulations and finds resonance in empirical evidence. Research has shown that organic fertilizers sourced from decomposed organic waste, plant residues, or animal byproducts significantly elevate the organic content of the soil and optimize it for sustained agricultural productivity (Shaji et al., 2021). Aside from being ecologically friendly, these products have demonstrated their advantages in enhancing soil texture, water retention, and microbial life (Fahrurrozi et al., 2019; Liu et al., 2022a; Liu et al., 2022b). Such soil improvements are pivotal forming a bedrock upon which healthier and more resilient crops thrive. Diverse in their constitution, organic fertilizers encompass a wide spectrum, from compost, a ubiquitous byproduct of decay, to more specialized derivatives, such as blood meal, fish emulsion, and seaweed extracts. Notably, advancements in the organic realm, such as the advent of controlled-release fertilizers (CRFs), have enabled sustained nutrient delivery merging the advantages of both organic and inorganic fertilizers. These fusions promise both nutrient enrichment and the reduction of environmental hazards (Shaji et al., 2021). However, the merits of organic nutrients have not been concealed. As reported by Ahmed et al. (2021b), crops cultivated using organic fertilizers, such as strawberries, often surpass their conventionally grown counterparts in terms of growth, nutrient content, taste, and longevity in shelf life. This quality enhancement is not an isolated incident; numerous studies have corroborated the superior nutrient profiles and health benefits associated with organically grown produce. However, the most resonant attribute of organic nutrients lies in their contribution to sustainable agricultural models. By championing biodiversity, these nutrients foster a vibrant soil microbiome that is integral for disease resistance and optimal nutrient uptake (Nikolaou et al., 2023). Furthermore, the incorporation of organic waste products, such as compost and manure, epitomizes the principles of a circular economy, minimizes waste, and ensures that natural cycles are respected and replicated.

### 6.2 The role of slow-release fertilizers in plant growth

A strong start in the early seedling phase not only ensures robust growth but also bolsters resistance against potential challenges. Slow-release fertilizers (SRFs) have emerged as pivotal players in ensuring that this critical phase of plant growth is optimally supported. One of the most innovative contributions to the annals of farming is that SRFs provide a nuanced understanding of plant nutrition. Unlike conventional fertilizers that offer a rapid, often excessive, dose of nutrients, SRFs gradually release these vital components. This modulated release, achieved through sophisticated encapsulation mechanisms or microbial degradation, ensures that plants receive a steady flow of nutrients (Nardi et al., 2018; Shaji et al., 2021). Such a sustained nutrient supply is instrumental in fostering the development of a robust root system, which is pivotal for the overall health and resilience of plants in later stages (Wang et al., 2020). The inception of nanofertilizers, particularly formulations, such as coated nanourea, accentuates the fusion of technology with traditional farming practices (Sharma et al., 2022). The observed enhancement in parameters, such as root and shoot length, photosynthetic pigments, and antioxidative capabilities in plants treated with nanourea/chitosan nanocomposites, as opposed to bare nanourea or commercial urea, indicates the potential of these novel fertilizers. However, their efficacy is intricately tied to the dosages applied, underscoring the need for precision in deployment. SRFs, while replete with their advantages, are not solutions. Their efficient utilization demands a comprehensive understanding of their interactions with environmental variables, compatibility with irrigation systems, and potential long-term impacts on soil health. However, the rewards, particularly for the early seedling stage, were significant. Not only do they sidestep the pitfalls of over-fertilization but their controlled release also ensures optimal nutrient assimilation laying the foundation for healthier, more vigorous crops (Fertahi et al., 2021). In the grand tapestry of sustainable agriculture, SRFs represent a thread of innovation, weaving scientific advancements together with age-old agricultural wisdom. As the push for sustainable, efficient, and eco-friendly farming practices has gained momentum, the role of slow-release fertilizers in nurturing the future, one plant at a time, is poised to be pivotal.

## 7 Pest management

### 7.1 The imperative of integrated pest and disease management

Agriculture is at a crossroads; on one hand, there is pressure to increase production to feed a growing global population; on the other hand, there is an urgent need to adopt sustainable practices to protect our planet and ensure that future generations can meet their needs. This delicate balancing act is evident in how pests and diseases that threaten crops are dealt with. Historically, the agricultural sector has heavily leaned from chemical pesticides. Their efficacy in decreasing pests and ensuring consistent yields has made them popular choices. However, over the years, environmental degradation and health risks associated with their overuse have become apparent (Lalruatsangi, 2021). Nurseries, which are vital to the agricultural supply chain, are particularly sensitive to these challenges. As breeding grounds for future crops, they require vigilant protection from pests and diseases; however, the methods used must not compromise plant health or the surrounding environment (Yadav et al., 2018). This is where IPM is performed. It is not just a method; it is a philosophy that integrates diverse agricultural practices ensuring that they work in tandem to control pests while minimizing environmental impact (Nayak et al., 2021). The true genius of IPM lies in its adaptability and reliance on a deep understanding of its ecological relationships. One of the core tenets of IPM is the use of biological controls essentially leveraging nature’s own mechanisms to control pests. From ladybugs to beneficial nematodes, these natural warriors help maintain balance ensuring that pests do not infest crops (Sasanelli et al., 2021). Yellow light has been widely studied for its effectiveness in controlling insect pests. Research has shown that yellow light traps are effective in capturing a variety of insect pests, including rice plant pests, tea green leafhoppers, and tomato leaf miners (Shimoda and Honda, 2013; Pezhman and Saeidi, 2018; Shi et al., 2021). The effectiveness of yellow light traps in capturing and monitoring insect pests has been a subject of interest in IPM strategies offering a non-invasive and environmentally friendly approach to pest control (Reddy, 2011). These studies collectively demonstrate the potential of yellow light traps as a valuable tool in the management of insect pests in agricultural settings.

Moreover, IPM stresses the importance of non-chemical interventions, such as crop rotation, intercropping, and the use of pest-resistant varieties (**Figure 3**). These methods not only deter pests but also enrich the soil and promote biodiversity (Paudel et al., 2020). However, for chemical interventions, IPM adopts a pragmatic approach. Chemicals are not entirely off the table, but are used sparingly, selectively, and only when other methods prove insufficient. This approach minimizes the risk of pesticide residues in food and the environment (Paudel et al., 2020). An exciting aspect of IPM is the potential of botanical pesticides. Plants, such as neem, garlic, and tobacco, have traditionally been used in various cultures to prevent pests. With modern research validating many of these traditional practices, there is renewed interest in harnessing their potential in a systematic manner. Not only are these plant-based interventions effective, but they also reduce the environmental footprint of agriculture (Lalruatsangi, 2021). As the world becomes increasingly aware of the ties between farming, well-being, and our planet, IPM stands as a guiding light. By merging scientific insights into age-old practices and environmental understanding, IPM charts the way toward a sustainable agricultural horizon.

### 7.2 Biological control agents and their role in IPM

The significance of IPM has been steadily gaining traction in the evolving landscape of agricultural and horticultural practices. Central to this is the concept of biological control, a practice that offers a more sustainable and environment-friendly alternative to the extensive use of chemicals (Jeffers and Chong, 2021). Entomopathogenic fungi (EPF) have emerged as one of the forerunners of non-chemical insect control. Their modus operandi revolves around acting as biological control agents presenting a potent combination of cost effectiveness, promotion of biodiversity, and a minimal environmental footprint. Furthermore, EPFs have the innate ability to inhabit plants as endophytes. This dual role, where they act both as agents of pest and disease control, and as promoters of plant growth, places them at the forefront of cutting-edge IPM strategies (Mantzoukas and Eliopoulos, 2020). The cultivation of flowering dogwood (*Cornus florida* L.) presents an illustrative example of the challenges faced and potential solutions offered by biological control. Powdery mildew disease is a significant concern. However, the discovery of the endophytic bacterium IMC8 offers a glimmer of hope. Demonstrating resilience across a range of conditions and compatibility with conventional fungicides, IMC8 stands out as a promising contender for powdery mildew. Through its production of volatile compounds with antifungal properties and evident parasitic activity against mildew, this bacterium exemplifies innovative and effective biological control solutions (Rotich et al., 2020).

Exploration of indigenous microbial communities in aquaponics as potential biocontrol agents offers a promising avenue for future research (Folorunso et al., 2021). In the diverse world of biological control agents, one encounters a range of organisms, each offering unique methods to combat pests. Predators, such as lady beetles and lacewings, actively hunt and reduce pest populations. In contrast, parasitoids, such as braconid wasps, employ more nuanced techniques. There are pathogens and competitors, exemplified by organisms, such as *Bacillus thuringiensis* (Bt), which challenge pests by introducing diseases or outcompeting them for resources. The overarching benefits of biocontrol span from environmental to economic. However, it is essential to acknowledge challenges, including ensuring successful establishment, potential side effects on non-target organisms, and navigating variable environmental conditions (Desurmont et al., 2018; Hewlett et al., 2019; Rotich et al., 2020; Nechols, 2021). The adoption and adaptation of biological control agents in IPM strategies represent a paradigm shift in modern agricultural practices. While the promise of a sustainable and environmentally conscious future is palpable, it is imperative to approach this transition with comprehensive research, informed understanding, and thoughtful implementation.

## 8 Plant growth regulators

Plant growth regulators (PGRs), whether naturally occurring or synthetic, play a transformative role in botany and horticulture profoundly impacting various stages of plant growth and development (Soni et al., 2022; Asghar et al., 2023). These potent compounds, categorized into older groups, such as auxins, cytokinins, gibberellins, abscisic acid, and ethylene, and newer groups encompassing hormones, such as jasmonates, salicylic acid, brassinosteroids, and polyamines, have been observed to have widespread applications in amplifying crop production. When applied judiciously, even in small amounts, PGRs can induce rapid phenotypic alterations in plants influencing their growth trajectory from germination to senescence (Asghar et al., 2023).

### 8.1 The role of plant growth regulators in vegetable farming

In the intricate choreography of vegetable farming, PGRs have emerged as pivotal players of both natural and synthetic origins (Soni et al., 2022). Their ability to invoke rapid phenotypic changes, even in minute quantities, and their profound influence on plant growth stages from germination to senescence, have garnered significant attention among vegetable farmers (**Figure 3**). Vegetable farming, a sector perpetually searching for enhanced productivity and quality, has especially benefited from PGRs. As highlighted by Soni et al. (2022), their applications span a wide range from seed soaking to inflorescence spraying. Furthermore, they are instrumental in hybrid seed production, improving seed germination vigor, and enhancing resistance against pests, diseases, and adverse growth conditions ultimately resulting in both qualitative and quantitative yield enhancements. A cornerstone of this growth narrative is the gibberellins, particularly noted for their role in breaking seed dormancy. This translates to accelerated seed germination, which is vital for vegetable seedlings and their subsequent timely transplantation into fields. Additionally, GA_3_-primed seedlings showed elevated resilience with improved antioxidant defense mechanisms facilitating better survival under stressors, such as drought (Zhang et al., 2022). Auxins, another subset of PGRs, are indispensable for vegetable cultivation because of their ability to foster robust root initiation, a fundamental requirement for healthy seedlings destined for transplanting. They not only stimulate root growth but also, as recent molecular insights suggest, influence embryonic fate in plants, enriching crop outcomes (Asghar et al., 2023). Although the primary focus of PGRs in vegetable farming has been growth promotion, it is pivotal to recognize their multifaceted roles. For instance, abscisic acid (ABA) empowers crop plants with enhanced drought resistance and regulates stomatal dynamics, a crucial adaptation for nurseries in arid regions or during water-scarce periods (Chen et al., 2020). However, vegetable farming demands a thorough understanding of the nuanced effects of PGRs. Despite its natural presence in plants, ethylene can expedite leaf senescence, which may not always be desirable in a vegetable farming system (Peerzada and Iqbal, 2021). Hence, precision in the application and awareness of environmental contexts is vital. Over-application can lead to toxicity, and staying abreast with regulatory guidelines is essential. For optimal vegetable farming and enhanced growth leading to better yields, a thorough grasp and wise use of PGRs, as highlighted by research such as that by Soni et al. (2022) and similar studies, is crucial.

### 8.2 Innovations in the application of PGRs for enhanced crop growth

In recent years, the methods used to apply plant growth regulators (PGRs) in agriculture have undergone revolutionary changes spurred by advancements in technology and deeper botanical insights. Traditional methods, such as soil drenching and foliar sprays, which once dominated the industry, have given way to more sophisticated and effective techniques (Bista et al., 2022; Kuts et al., 2023). A breakthrough in this field was demonstrated in the study of onions. When specific concentrations of the growth regulators GA_3_ and NAA were applied at precise stages of the onion life cycle, there was a marked enhancement in attributes such as plant height, number of leaves, and bulb diameter. Specifically, a regimen of 150 mg L^−1^ of NAA at the three-leaf stage and 150 mg L^−1^ of GA_3_ at the seven-leaf stage yielded the most promising results (Bista et al., 2022). In line with these discoveries, Kuts et al. (2023) discussed the potential of both synthetic and organic PGRs in modern vegetable cultivation. In particular, they underscored their role in increasing productivity, enhancing product quality, and bolstering resilience against environmental challenges. For instance, when cucumbers grown in film greenhouses are treated with specific growth regulators and microfertilizers at various stages, there is a significant increase in fruit yield.

Seed priming is central to these innovative application methodologies. PGR-rich solutions were used to soak the seeds prior to sowing. This resulted in increased germination rates and enhanced root vigor. This priming has shown remarkable effects on pepper seeds. A factorial experiment indicated that priming seeds with specific concentrations of GA_3_ and NAA significantly improves the germination and growth characteristics of various pepper cultivars (Tombegavani et al., 2020). A further innovation frontier is the creation of PGR-embedded slow-release pellets. These pellets utilize materials, such as carboxymethyl cellulose, chitosan, and polylactic acid, to encapsulate PGRs ensuring a slow and steady release of these crucial growth enhancers to plants (Badgar et al., 2022). Complementing this is the rise in nanoencapsulation. By encapsulating PGRs in nanoscale carriers, these growth agents are protected from premature degradation, thereby providing crop plants with a longer supply of nutrients (Zaim et al., 2023). Technological advances, such as electrostatic spraying, have also been incorporated to ensure the even and precise application of PGRs (Zaim et al., 2023). In tandem with these developments, biostimulants, although distinct from traditional PGRs, show promise for augmenting plant growth. Another significant leap is the aeroponic method, wherein PGRs are incorporated into nutrient mists showing the extent to which technological innovation has been interwoven into horticulture. However, while these advancements offer numerous benefits, they also face challenges particularly in terms of the costs and expertise required for their application. Despite these hurdles, the overarching consensus suggested by Sajjad et al. (2017) is that the future of PGR applications in agriculture is bright, with these innovative techniques poised to significantly elevate crop health and productivity.

## 9 Technological innovations in vegetable cultivation

**Figure 4:**
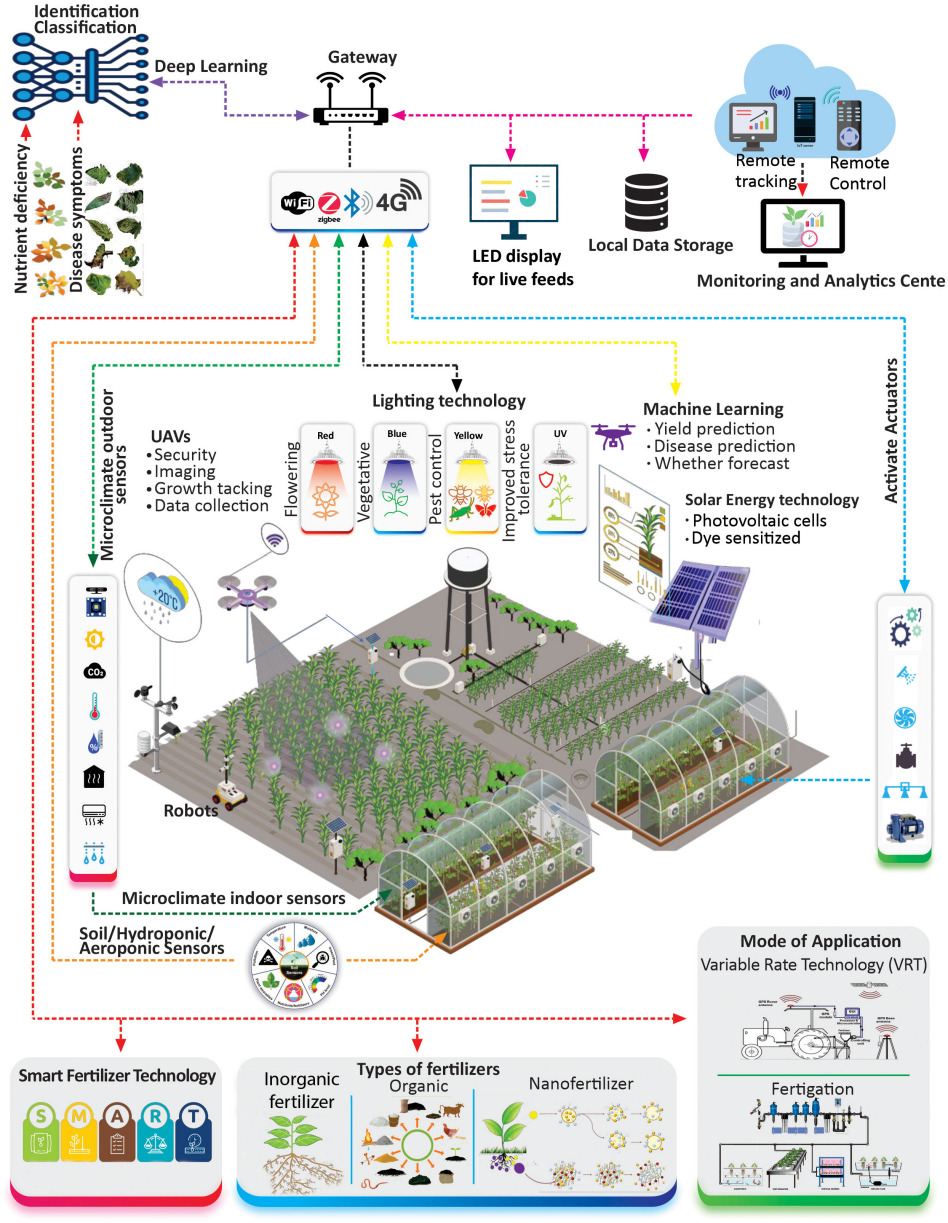
This illustration encapsulates the integration of cutting-edge technologies into modern greenhouse vegetable farming. From aerial monitoring by drones to the depth of soil microbiomes, each advancement, represented by nodes, collaboratively optimizes plant growth, health, and productivity. Seamless synergy between natural processes and innovative technologies illuminates the future of sustainable and precise agriculture.

The dawn of the 21st century has witnessed a transformative phase in vegetable cultivation spurred by groundbreaking technological innovations. Advanced imaging techniques for meticulous crop monitoring (Ampatzidis and Partel, 2019) with the introduction of drones and robotics ensure precision in operations (Warner et al., 2022). The integration of modern technologies reshapes the fabric of horticultural practices (**Figure 4**). Furthermore, as greenhouses harness the prowess of automation for optimal environmental control (Shamshiri et al., 2018b), the promise of a sustainable and efficient agricultural future becomes apparent.

### 9.1 Harnessing rainwater and solar energy for sustainable growth

In the dynamic domain of horticulture, contemporary greenhouses have transformed from mere sunlight enclosures to epitomes of avant-garde technology and stringent environmental regulations (Koukounaras, 2021). These facilities have transcended their passive origins and evolved into responsive hubs designed to meet diverse plant requirements. Embodying the attitude of sustainability, modern greenhouses have championed initiatives, such as rainwater harvesting and cyclical water use, fortifying their ecological credentials (Oweis, 2022). The linchpin of this metamorphosis is the fusion of progressive technologies with judicious environmental management. A paramount example is a dye-sensitized solar cell (DSSC). These cells adeptly reconcile energy consumption with optimal sunlight dispersion making their niche an economical and versatile solution in an era sensitive to energy utilization (Koukounaras, 2021). Unlike some traditional solar technologies that inadvertently impede plant growth within greenhouses, DSSCs ingeniously modulate the solar input to favor plant development (**Figure 4**).

### 9.2 LED technology and automation: revolutionizing horticultural productivity and sustainability

In recent decades, LED technology has been at the forefront of lighting innovation in plant cultivation. Automated lighting systems are equipped with daylight sensors that gauge natural light levels and supplements, as needed (Barceló-Muñoz et al., 2022). Advanced LED systems allow spectrum control tailored to specific growth stages such as blue light for vegetative growth or red light for flowering (**Figure 4**) (Kim et al., 2020). Moreover, automatic adjustments protect plants from light stress ensuring that they receive optimal illumination (Paradiso and Proietti, 2022). Kim et al. (2020) highlighted the potential of intra-canopy LEDs in improving the quality and yield of off-season tomatoes. Compared with traditional high-pressure sodium (HPS) lamps, tomatoes grown under specific LED wavelengths exhibited improved physicochemical attributes and increased mineral content. Moreover, LEDs have proven to be more energy efficient underscoring their value in modern agriculture. On the sustainable innovation front, technologies, such as dye-sensitized solar cells and advanced LED lighting, as advocated by Koukounaras (2021), suggest a harmonious blend of productivity enhancement while curbing agriculture’s environmental footprint. Drawing parallels, the research presented by Shadrin et al. (2019) on the niche of vegetable farming exhibits similar trends.

### 9.3 The role of automation in modern greenhouse cultivation: climate control and CO_2_ enrichment

Advancements in greenhouse technologies have dramatically altered the plant cultivation landscape particularly through the integration of automation in climate control. Automated systems, including ventilation, air conditioning, and lighting, are tailored to meet the specific requirements of different plant species (Shamshiri et al., 2018b; Hemming et al., 2019). For instance, automated ventilation systems with roof and side vents respond dynamically to changes in temperature and humidity ensuring optimal airflow necessary for plant health (Fu et al., 2023). Variable-speed fans also contribute to adaptable cooling and air circulation aligning with the current microclimate needs (Hemming et al., 2020). In regions with extreme weather conditions, air conditioning systems play a crucial role in providing precise control over temperature and humidity, which is essential for maintaining the desired levels of CO_2_ and other gases within the greenhouse (Colantoni et al., 2018). Solar greenhouses exemplify the synergy between natural ventilation and automated climate control, where the balance between internal and external environments is vital for energy efficiency (Chen et al., 2018; Fu et al., 2023). The interaction between outdoor wind speeds and automated vent openings is essential for achieving desired ventilation rates and regulating indoor temperatures (Chen et al., 2018; Jiang et al., 2023). Additionally, sophisticated automated systems, such as heat pumps and radiant floor heating, modulate their output based on real-time climate data to sustain ideal growth temperatures while enhancing energy conservation efforts (Han et al., 2022; Fu et al., 2023). These precise climate control mechanisms are indispensable not only for warmth but also for managing the heat generated by in-greenhouse equipment, which can collectively contribute to overheating if not adequately regulated. Furthermore, the strategic implementation of CO_2_ enrichment has become a cornerstone of modern greenhouse automation. Advanced monitoring systems ensure that CO_2_ concentrations are kept at optimal levels enabling plants to maximize their photosynthetic potential while conserving CO_2_ resources (Gao et al., 2010; Achour et al., 2020; Huber et al., 2021). Collectively, these technological innovations signify a shift from manual interventions to automated solutions that not only reinforce sustainable farming practices but also enable precise interventions for various plant requirements.

### 9.4 The emergence of precision fertilization techniques

Precision fertilization, blending technology, and scientific expertise offer innovative solutions to the challenge of harmonizing sustainable agricultural practices with enhanced yields (**Figure 3**). The core of this approach lies in soil testing ensuring that soil health parameters are understood and interpreted to drive effective fertilization (Mikula et al., 2020; Podar and Maathuis, 2022). Building on this foundation, variable-rate technology (VRT) integrates GPS systems charting the way for tailored fertilization strategies that can vary within a single field (Kumar and Nirosha, 2023). Such granularity in nutrient management is further emphasized through fertigation and foliar fertilization, both of which present avenues for direct nutrient delivery resulting in efficient fertilizer use and rapid responses to nutrient deficiencies (Ahmad et al., 2018; Bar-Yosef, 2020). Furthermore, controlled-release fertilizers (CRF) gain traction, and their ability to maintain calibrated nutrient release emerges as a boon ensuring that plants receive a balanced nutrient supply while simultaneously reducing potential environmental impacts (Cole et al., 2016). Remote sensing and drone technologies are poised to reshape precision agriculture. The exceptional spatial resolution and data-capture capabilities of unmanned aerial vehicles (UAVs) offer real-time insights enabling farmers to make immediate and informed decisions (Maes and Steppe, 2019). Soil spectroscopy is a technique that uses infrared technologies to swiftly gauge soil properties allowing for more accurate fertilization (Salimi and Boelt, 2019; Mahmud et al., 2023). Today, farmers also have digital allies in the form of Smart Fertilizer Management (SFM) software. These platforms amalgamate data from diverse sources, from soil samples to satellite imagery, and craft fertilizer recommendations tailored to distinct fields (Agrahari et al., 2021). Innovations, such as nanofertilizers, developed via nanotechnology, promise enhanced nutrient efficiency owing to their ability to permeate plant cell walls more effectively than their traditional counterparts (Gomes et al., 2020). The soil microbiome also has potential as a resource. Beneficial microorganisms, such as certain bacteria, can either fix atmospheric nitrogen or render soil phosphorus soluble reducing the dependency on chemical fertilizers and steering agriculture toward more sustainable pathways (Tavarini et al., 2018; González-Guerrero et al., 2023). The combination of technology and biology is further evident in innovations such as *in situ* soil moisture sensors, 3D printed fertilizers tailored to specific crop needs, chlorophyll meters that gauge plant nitrogen levels, and decision support systems (DSS) that assimilate diverse data to guide fertilization decisions (Ahmad and Dar, 2020; Hemming et al., 2020; Gorai et al., 2021). In summary, it is clear that the trajectory of precision fertilization has been marked by rapid advancements. As global challenges surrounding population growth and environmental preservation intensify, these nuanced fertilization techniques stand as pillars for a sustainable agricultural future bridging the gap between high yields and responsible farming.

### 9.5 Advanced imaging and deep learning for pest management and crop monitoring

Advanced imaging and deep learning technologies have ushered in a revolution in agricultural pest management and crop health assessment. These innovative developments have combined the power of deep learning, including deep neural networks, with a range of imaging modalities, such as hyperspectral, RGB, multispectral, IR, and NIR, to reshape the landscape of agriculture (**Figure 3**) (Chandel et al., 2021; Liang et al., 2021; Abdullah et al., 2023). This integration has given birth to innovative solutions for pest detection, classification, and localization leading to a significant improvement in agricultural efficiency. Deep learning models have been extensively applied for the detection and diagnosis of plant diseases and pests offering promising results and large potential in image processing and data analysis (Ferentinos, 2018; Kamilaris and Prenafeta-Boldú, 2018; Liu and Wang, 2021), These models have been particularly effective in early pest detection, such as the recognition of insect pests at the larval stage before planting, allowing for precise localization and targeted intervention to minimize chemical usage (Obasekore et al., 2023). This early identification allows for precise localization and targeted intervention minimizing the need for chemical pesticides. Moreover, the accuracy and reliability of pest detection have been significantly enhanced by deep learning technology. Ensemble models based on deep learning have demonstrated the ability to detect anomalies with remarkable performance (Madhavi et al., 2021; Lee et al., 2022). This means that farmers can rely on these models for dependable and timely pest detection reducing the risk of crop damage.

The integration of advanced imaging and deep learning technologies with UAVs has further expanded their impact on agri-pest management. UAVs have become indispensable tools for providing expansive mapping and precise monitoring of agricultural fields. Deep learning-based visual recognition, when paired with UAVs, has enabled the identification of pests and diseases with exceptional accuracy (Tsouros et al., 2019; Ecer et al., 2023). Additionally, the conjunction of deep learning with UAVs has given rise to mobile applications for real-time insect pest detection. These applications consolidate image collection, data preprocessing, and modeling strategies streamlining agricultural practices (Doan, 2022). For example, improved YOLO V3 convolutional neural networks have enabled the high-precision detection of tomato diseases and pests (Liu and Wang, 2020). Similarly, deep learning-based methods for light-trap pest detection have addressed the challenge of varying object numbers and size distributions resulting in improved performance (Teng et al., 2022). A comprehensive survey of recent studies highlights the potential of UAVs in precision agriculture further emphasizing their significance in modern farming practices (Aslan et al., 2022). The integration of advanced imaging modalities with UAVs has contributed to large-scale mapping and enhanced agricultural efficiency. These technologies are not only limited to pest detection but also extend to disease diagnostics and non-destructive methods such as fluorescence imaging.

The development of crop monitoring methods has been significantly influenced by the role of multispectral and hyperspectral imaging in detecting subtle changes in plant health. Hyperspectral imaging, in particular, has proven instrumental in the early detection and classification of plant diseases (Kong et al., 2018; Nagasubramanian et al., 2019). These studies have underscored the potential of hyperspectral imaging as a fast, non-destructive, and reliable technique for disease detection on plant stems. Furthermore, the application of deep learning on hyperspectral images has shown promise in detecting diseases such as potato virus Y in seed potatoes (Polder et al., 2019) and *Aphis gossypii* Glover infection in cotton leaves (Yan et al., 2021). This highlights the profound impact of advanced imaging techniques on disease identification in agriculture. The integration of hyperspectral imaging with remote sensing has opened up new possibilities for detecting and analyzing weed infestations in rice fields (Sulaiman et al., 2022). This application demonstrates the potential of hyperspectral remote sensing imagery in weed detection and analysis contributing to improved crop management practices. Chlorophyll fluorescence imaging has been a valuable tool for capturing the photochemical efficiency of grain sorghum in field settings offering insights into plant photosynthesis and health (Herritt et al., 2020). Additionally, the detection of citrus huanglongbing in Brazilian orchards using hyperspectral aerial images has shown the potential of hyperspectral imaging in identifying and monitoring diseases in agricultural settings (Moriya et al., 2019). These advancements have significantly contributed to our understanding of plant health, disease diagnostics, and crop management ultimately impacting modern agricultural practices and efficiency.

The integration of advanced imaging technologies with unmanned aerial vehicles (UAVs) and AI/ML algorithms has further enhanced their impact on modern agricultural practices. This combination enables rapid and non-invasive characterization of plant health, disease detection, and improved crop management. As documented by Ampatzidis and Partel (2019), this technique offers an early detection system that captures subtle shifts in plant health. Thermal imaging has proven effective in shedding light on plant transpiration dynamics and water-related stress (Shoa et al., 2022). Fluorescence imaging, as described by Valcke (2021), acts as a sentinel for photosynthetic efficiency, flagging potential stressors indicated by fluorescence deviations. Stereoscopic imaging and light detection and ranging (LIDAR) provide spatial insights on plant biomass and terrain (Wiering et al., 2019) facilitating superior drainage systems and optimal layout configurations for crop growth. Simultaneously, X-ray imaging offers a promising modality for meticulous seed quality assessments ensuring that farmers have access to defect-free seeds (de Medeiros et al., 2021). To complete the imaging spectrum, RGB imaging, championed by Mahmud et al. (2023), offers direct monitoring capabilities when integrated with analytical platforms. This simple, yet effective, method provides a clear-cut approach to overseeing growth dynamics and preempting potential challenges. The integration of these technologies with unmanned aerial vehicles and AI/ML algorithms has significantly enhanced their impact on modern farming practices. These advancements not only offer early and precise pest detection but also contribute to large-scale mapping, disease diagnostics, and improved agricultural efficiency.

### 9.6 Use of AI, ML, IoT, drones, and robotics in vegetable farming

The integration of advanced technologies, including drones, robots, AI, ML, and the IoT, has revolutionized modern agriculture, particularly in the context of precision farming and vegetable cultivation. The adoption of drones in precision farming has seen significant growth, with continuous innovation and selective application of inputs driving this trend (Bai et al., 2022). Similarly, the development of scouting robots equipped with advanced sensors has enabled precise data collection and navigation control contributing to the automation of farming processes (Shamshiri et al., 2018a). Furthermore, the use of AI and ML algorithms in conjunction with IoT technologies has facilitated the implementation of smart agriculture systems leading to improved crop productivity and resource management (Alreshidi, 2019; Adli et al., 2023). In the realm of vegetable farming, precision technologies, cloud computing, and IoT have been harnessed to enhance crop productivity and optimize resource utilization (Kaushik, 2021). The application of AI and ML in smart agriculture has paved the way for the development of IoT-enabled systems for decision making and control resulting in improved crop management and reduced operational costs (Ramakrishnam Raju et al., 2022). Additionally, the integration of AI and IoT technologies has led to the design and implementation of smart hydroponic farming systems further demonstrating the potential for advanced technologies to enhance agricultural practices (Ramakrishnam Raju et al., 2022). The use of robotics in vegetable farming has also been a focal point with robots playing a critical role in preventing future food crises caused by population growth (Li et al., 2022). Furthermore, the integration of AI and IoT technologies has resulted in the development of smart agriculture cloud-based systems enabling remote monitoring and management of agricultural operations (Junaid et al., 2021). The incorporation of intelligent services and cognitive components in IoT architectures has modernized IoT infrastructures allowing for the seamless integration of AI technologies (Valero et al., 2021).

The convergence of AI, ML, and IoT in modern agriculture has not been without challenges. The sheer volume of data generated in smart agriculture systems presents a significant challenge necessitating robust big data analytics and management solutions (Hariri et al., 2019). Additionally, the security and interpretability of AI-based smart agriculture systems have been areas of focus highlighting the need for reliable and secure IoT and AI implementations in agricultural settings (Sabrina et al., 2022; Patel et al., 2023). The integration of drones, robots, AI, ML, and IoT technologies has paved the way for precision farming and vegetable cultivation resulting in enhanced crop productivity, resource management, and operational efficiency. These strides in smart agriculture systems highlight the immense transformative potential of advanced technologies in shaping the agriculture of tomorrow.

## 10 Sustainability in vegetable cultivation

The agricultural domain is undergoing a transformative shift with the pivot toward more sustainable and organic practices becoming a focal point. In this context, vegetable cultivation has emerged as a linchpin with the onus developing and maintaining vegetable crop health while minimizing ecological impact (**Table 1**). A burgeoning body of research has underscored this sentiment. One of the seminal works in this sphere is from Otero et al. (2019), who contended that sustainable farming practices are imperative to address the escalating ecological challenges faced by the horticulture sector. This emphasis on sustainability was buttressed by Tahat et al. (2020), who highlighted the virtues of organic growing media. According to their findings, these media not only bolster soil health but also curtail the reliance on depleting non-renewable resources. Adding a layer to this discussion, Rodriguez et al. (2021) compelled the integration of companion plants and cover crops into nurseries. Their research demonstrated that such integrations deter pests and augment soil nutrition consequently reducing the dependence on chemical interventions. Pest management, a perennial concern in horticulture, has been innovatively addressed through sustainable avenues. Nazir et al. (2019) offer an intriguing perspective on this by delving into biological pest control. These studies revealed that beneficial insects, including ladybugs and nematodes, can serve as natural deterrents against harmful pests. Such practices, while ensuring crop health, also safeguard the broader ecological balance. Sustainable materials have emerged as cornerstones of contemporary vegetable cultivation. For example, the proliferation of biodegradable pots, as documented by Cherian et al. (2022), presents a two-pronged advantage. Not only do these pots enhance soil decomposition, but they also significantly reduce plastic waste. Similarly, Li et al. (2021) shine a light on the rise of organic mulches emphasizing their role in moisture retention, weed suppression, and nutrient replenishment. However, the conversation regarding sustainability is incomplete and does not address the role of water. Here, the insights from Bafdal and Dwiratna (2018) stand. They underscored the potential of rainwater collection in nurseries, a practice that leads to marked reductions in water consumption and operational costs. This is complemented by the findings of Redekar et al. (2020) on recycled water systems, which emerge as a paradigm of conservation while ensuring an uncontaminated water supply for crops. Incorporating sustainable practices is not only related to ecological responsibility. Therefore, there is a pressing need to ensure knowledge diffusion for broad-based adoption. Kumar et al. (2018) highlight this very need advocating for comprehensive training programs tailored for farming personnel. The future, as envisioned by Bhandari and Nayama (2020), is one of the syntheses where traditional sustainable practices meld with cutting-edge technologies setting new benchmarks in eco-conscious horticulture. In sum, the trajectory of vegetable management is unequivocal pointing toward an intertwining of environmental stewardship with agricultural progress. The synthesis of research in this domain unequivocally suggests that sustainable vegetable farming is not merely a desirable path but is imperative for the holistic well-being of the global horticulture industry.

**Table 1:** Sustainable practices in vegetable cultivation.

Sustainability aspect	Practice	Benefits	Common implementations	References
Water conservation	Techniques designed to reduce water usage	Environmental responsibility; cost savings	Rainwater harvesting, drip irrigation	(Bar-Yosef, 2020; Oweis, 2022)
Organic practices	Avoiding synthetic chemicals in favor of natural solutions	Healthier plants; eco-friendly; market demand	Organic fertilizers, biopesticides	(Samada and Tambunan, 2020; Shaji et al., 2021)
Waste recycling	Reusing resources or converting waste into valuable products	Waste reduction; cost efficiency	Composting, mulching	(Zinati, 2005; Sari, 2022)
Renewable energy	Using renewable sources for powering farm operations	Reduce carbon footprint, energy savings; lower operational costs	Solar panels, wind turbines	(Roslan et al., 2018; Grant et al., 2022)
Bio-based growing mediums	Using sustainable, renewable resources as an alternative to traditional growing mediums	Environmental sustainability; improved plant health	Coir, biochar, composted bark, plant growth-promoting rhizobacteria	(López-López and López-Fabal, 2016; Mariotti et al., 2020; Tahiri et al., 2022)
Reduction in energy consumption requirements	Implementing energy-efficient technologies and optimizing resource allocation	Decreased operational costs, reduced environmental impact, enhanced sustainability	Use of solar panels, LED lighting for plant growth, smart sensors for precision agriculture, energy-efficient climate control systems in greenhouses	(Morrow, 2008; Akrami et al., 2020; Goel et al., 2022; Soussi et al., 2022)

## 11 Potential drawbacks and concerns with new approaches

Like many other sectors, the realm of vegetable farming is undergoing rapid transformation driven by technological advancements. While these innovations offer tremendous potential, they also face a suite of challenges. This review attempts to shed light on the multifaceted concerns that these advancements have brought to the fore.

**Environmental concerns**: *Resource overconsumption*: Modern systems, in their bid to maximize outputs, often demand high energy or water inputs. Although they might bolster productivity, they can inadvertently strain our already dwindling resources (Ghasemi-Mobtaker et al., 2020). *Waste generation* further aggravates environmental concerns. The infusion of non-biodegradable components into many new technologies escalates waste production especially when recycling options are scarce (Kibria et al., 2023).

**Biological and ecological concerns**: The realm of biology is immune to these concerns. *Overreliance* on a limited pool of genetically engineered plants threatens the biodiversity. This homogeneity can make crops vulnerable to diseases, thereby weakening the resilience of ecosystems (Caradus, 2022). Adding to ecological woes is the *chemical residue* left behind by synthetic growth promoters and pest control agents. These residues can harm beneficial organisms and have lasting repercussions for environmental health (Mateos Fernández et al., 2022).

**Economic and social concerns**: From economic standpoint, the risk of *market dependency* is large. Certain cutting-edge techniques might hinge on patented inputs, providing disproportionate market power to a handful of corporations. This scenario can spawn dependency and lead to monopolistic behaviors that impact pricing (Hussain et al., 2020). *Equity concerns* are paramount. The disparity between large-scale, affluent nurseries and their smaller, resource-constrained counterparts could widen because the former might have exclusive access to expensive high-tech solutions (Talukder et al., 2020).

**Technological concerns**: Technological integration, while impressive, presents its own set of challenges. The complex nature of new systems can compromise *reliability*. Malfunctions in such intricate setups can result in substantial losses (Mistry et al., 2020). Additionally, an *over-dependency* on technology can lead to the erosion of traditional skills. In scenarios where technology falters or is accessible, skill attrition can have significant consequences.

Although the allure of technological and innovative strides is undeniable, a circumspect approach is essential. It is vital to temper enthusiasm with caution to ensure that the broader implications of each advancement are thoroughly vetted. The cited studies offer deeper insights and provide a foundation for anyone seeking a profound understanding of these challenges.

## 12 Future prospects

The evolution of advanced vegetable cultivation practices has thus far been nothing short of being remarkable. Over the past decade, the integration of technology with traditional practices has sculpted the landscape of immense potential. While predicting the future with pinpoint accuracy is ambitious, the present trends and technological underpinnings offer illuminating insights.

### 12.1 Prediction of future advances in vegetable farming

**Genomic insights:** The roadmap of genomic research points to a future where the genetic blueprint of plants holds the key to transformative practices. The prospect of plant varieties tailored to regional idiosyncrasies, individual crop specifications, or particular soil types is becoming increasingly tangible (Bhowmik et al., 2021).

**IoT integration:** IoT is merely a buzzword, the backbone of the next industrial revolution. In the context of vegetable farming, imagine a world where real-time monitoring systems assessing variables, such as soil moisture or light levels—feed data that automatically modulate crop environments (Naik et al., 2021).

**VR and AR in training:** VR and AR revolutionize training paradigms. From in-depth anatomical explorations of plant structures to real-time disease diagnostics, these technologies can facilitate immersive learning experiences and transform novices into experts (Yousif, 2022).

**Decentralized AI-driven decisions:** The shift from centralized to decentralized (or edge) computing can redefine the response times in crop management. With decisions anchored at the device level, the latency in system responses can plummet making management more agile (Slob and Hurst, 2022).

**Circular economy in vegetable farming:** The call for sustainability is answered with the circular economy model. Its ethos of resource conservation, emphasizing the loop of reuse, recycling, and upcycling, will steer vegetable cultivation toward unprecedented sustainability milestones (Zarbà et al., 2019).

**Personalized plant care:** The paradigm of personalized care is confined to human medicine. Precision horticulture, built on the premise of customizing care to individual plant needs, can elevate plant health and optimize resource allocation (López et al., 2009; Atreya et al., 2019).

Essentially, the future trajectory of vegetable farming sparkles with a blend of traditional knowledge and avant-garde innovations, amplifying sustainability, and efficiency. However, the path is speckled with challenges in navigating ethical conundrums discerning economic implications and ensuring the reliability of emerging technologies. Progression in this sector will hinge on an equilibrium between empirical research and informed judgment paving the way for a thriving nexus between flourishing plants and evolved practices.

### 12.2 The role of research and development in orchestrating vegetable cultivation renaissance

In the vast tapestry of industrial evolution, research and development (R&D) has emerged as a silent weaver stitching together the complex interplay of science, technology, and market demand. Vegetable farming, a field where nature meets nurture, finds R&D an ally invaluable. This is a deeper exploration of the transformative role of R&D.

1. **Bridging practical and theoretical knowledge:** The dynamism of R&D transforms theoretical paradigms into practical solutions. It acts as a conduit, ensuring that laboratory breakthroughs are confined to scholarly papers, but find resonance in the soil and seeds of nurseries.
2. **Tailored solutions for specific challenges:** One-size-fits-all is an anachronism in modern crop cultivation practices. R&D, with its deep dive into regional idiosyncrasies, crafts solutions that address unique challenges, such as the saline soils of coastal regions or drought-prone terrains of the hinterlands.
3. **Integration of advanced technologies:** R&D is the crucible in which the technology mettle was tested: the evolution of drone-based monitoring systems, IoT-integrated irrigation models, and AI-driven pest prediction matrices, and their R&D that catalyze their journey from prototypes to practical solutions.
4. **Sustainable and organic innovations:** As consumers’ palates become discerning, prioritizing organic over synthetic, R&D shoulders the responsibility of developing eco-friendly yet effective vegetable farming strategies. This entails research on sustainable pesticides, eco-friendly fertilizers, and holistic farming practices.
5. **Improved seed varieties:** At genetic frontier, R&D deciphers the code of life leading to the genesis of seed varieties that can weather adversities, resist diseases, and flourish even in challenging environments.
6. **Skill development and capacity building:** Beyond tangible aspects, R&D has an educational facet. It nurtures talent, equipping the custodians of nurseries with skills that marry traditions with technology, ensuring the industry’s vibrancy and relevance.
7. **Feedback loop creation:** R&D is not a monolog; it thrives on dialog. The feedback loop it establishes ensures that research is not conducted in silos but is continually refined based on ground-level insights and real-world challenges.

To encapsulate, R&D is a compass that ensures that vegetable farming does not meander but marches forward with purpose and clarity. It is the harbinger of a future where vegetable cultivation farm are not spaces of crop cultivation but cradles innovation. For the industry to flourish, its stakeholders must not just apply R&D from the sidelines, but must actively champion and invest in it, recognizing its unparalleled role in shaping tomorrow.

## 13 Conclusion

In the present milieu, vegetable cultivation practices are on the brink of significant metamorphosis. This comprehensive review intricately traversed the various layers of this transformation ranging from the delicate intricacies of seed choice to wider frameworks centered on sustainability and impending advancements. The forefront of our discourse is several pivotal insights. The fusion of genomics with advanced evaluation strategies suggests a promising path for cultivating both resilient and prolific crops. There has been a discernible shift in foundational vegetable farming practices highlighted by the rise of soilless cultures and advanced environmental modulating techniques. Water and nutrition management now underlines precision and sustainability reflecting a sector-wide inclination toward optimal resource stewardship. Current trends in pest management and growth regulation have revealed a diminishing reliance on chemical means paralleled by inventive strategies that promise greater agricultural output. Furthermore, the merger of traditional farming practices with emerging technologies, such as drones and AI, sketches the portrait of a rapidly transforming vegetable cultivation landscape. The move toward environmentally responsible measures complements an invigorating future painted with the strokes of genomics, the IoT, and AI. However, anchoring these progressive deliberations is an unwavering emphasis on solid research and development foundations. Beyond these specific insights, a more expansive theme becomes evident: the non-negotiable need for relentless research and fluid strategy. In a realm as dynamic as vegetable farming, where challenges such as ever-evolving pests and shifting climatic norms are given, the crux lies in persistent research and adaptability. By navigating new obstacles to embracing technological breakthroughs, prioritizing sustainability, and maximizing yield, these two facets stand out as central tenets. In conclusion, vegetable cultivation practices reflect a delicate balance between age-old practices and modern paradigms. While the journey is rife with challenges, these are set against a canvas teeming with potential. For those vested in this domain, the mandate is clear: invest in learning, celebrate flexibility, and gear up for a horizon rich in both trials and triumphs, which are all crucial to ensuring global food stability.

## Author contributions

NA: Conceptualization, Formal analysis, Investigation, Visualization, Writing – original draft, Writing – review & editing. BZ: Conceptualization, Writing – original draft, Writing – review & editing, Resources, Validation. LD: Formal analysis, Investigation, Writing – review & editing. BB: Software, Visualization, Writing – review & editing. JL: Conceptualization, Formal analysis, Writing – review & editing. SC: Investigation, Methodology, Writing – review & editing. ZC: Formal analysis, Investigation, Writing – review & editing. IJ: Investigation, Visualization, Writing – review & editing. AT: Investigation, Validation, Writing – review & editing. MG: Investigation, Resources, Writing – review & editing. FH: Formal analysis, Investigation, Writing – review & editing. PT: Funding acquisition, Project administration, Resources, Supervision, Writing – review & editing.
